# P2Y1R Ligation Suppresses Th17 Cell Differentiation and Alleviates Colonic Inflammation in an AMPK-Dependent Manner

**DOI:** 10.3389/fimmu.2022.820524

**Published:** 2022-02-10

**Authors:** Yao-Yao Chang, Qiu-Chan Huan, Jiao Peng, Wen-Chun Bi, Li-Xiang Zhai, Yan Chen, Jonathan R. Lamb, Xiang-Chun Shen, Zhao-Xiang Bian, Hai-qiang Wu, Yong-Xian Cheng, Hai-Tao Xiao

**Affiliations:** ^1^ School of Pharmaceutical Sciences, Health Science Center, Shenzhen University, Shenzhen, China; ^2^ The State Key Laboratory of Functions and Applications of Medicinal Plants and The High Efficacy Application of Natural Medicinal Resources Engineering Center of Guizhou Province, School of Pharmaceutical Sciences, Guizhou Medical University, Guizhou, China; ^3^ Department of Pharmacy, Peking University Shenzhen Hospital, Shenzhen, China; ^4^ School of Chinese Medicine, Hong Kong Baptist University, Kowloon, Hong Kong SAR, China; ^5^ Department of Surgery, The University of Hong Kong, Hong Kong, Hong Kong SAR, China; ^6^ Department of Life Sciences, Faculty of Natural Sciences, Imperial College London, London, United Kingdom

**Keywords:** P2Y1R, Th17 cell, differentiation, colitis, AMPK

## Abstract

P2Y1 receptor is a G-protein-coupled receptor that plays a critical role in the immune response of inflammatory bowel diseases. However, its regulatory effects on CD4^+^ T cell response have not been fully elucidated. The study aimed to characterize the role of P2Y1R in Th17 cell differentiation and colonic inflammation. Our results demonstrated that P2Y1R was significantly increased in the splenocytes of colitic mice, which was positively associated with the expression of RORγt and IL-17A. P2Y1R deficiency significantly ameliorated DSS-induced colitis and its Th17 responses. In parallel, P2Y1R deficiency greatly impaired the differentiation of Th17 cell, down-regulated the mRNA expression of IL-17A and RORγt, and protein expression of RORγt *in vitro*. More importantly, it was found that P2Y1R deficiency markedly increased AMPK phosphorylation of Th17 polarized CD4^+^ T cells, and antagonist of AMPK significantly reversed the inhibitory effect of P2Y1R deficiency on Th17 cell generation *in vivo* and *in vitro*. Overall, these findings demonstrated that P2Y1R deficiency could suppress Th17 cell differentiation in an AMPK-dependent manner to ameliorate colitis, and P2Y1R can act as an important regulator of Th17 cell differentiation to control colonic inflammation.

## Introduction

Inflammatory bowel disease (IBD) is a chronic immune-mediated inflammatory disease that is difficult to cure and easy to relapse, which includes Ulcerative colitis (UC) and Crohn’s disease (CD). Although the precise pathogenesis of IBD remains incompletely understood, studies indicated that excessive pathogenic Th17 cells in the colon is the root of evil for the start and development of IBD (1–4). Notoriously, Th17 cells are a subset of effector T cells distributed in the mucosa, mostly in intestinal mucosa, which usually secret inflammatory cytokines such as IL-17A, IL-21 and IL-23 to injury colon tissues (5, 6). It is reported that the number of Th17 cells, gene and protein expression of Th17 cell-specific inflammatory cytokines like IL-17A, IL-21 and IL-23 were significantly increased in the inflamed intestinal mucosa in comparison of uninflamed sites (7–9). Additionally, the evidence from animal models also showed that Th17 cells transferred to immunodeficient Rag1^-/-^ mice induced severe colitis (10). Accordingly, it is of great significance to investigate the regulatory mechanism of Th17 cells for further discovering the potential pathogenesis and therapeutic target of IBD.

During process of intestinal inflammation, in response to pathogenic microbial infection and cell injury, intestinal cells including endothelial cells, mast cells, and macrophages release a lot of nucleotides such as adenosine triphosphate (ATP) and adenosine diphosphate (ADP), which are accumulated at sites of inflammation and function as danger signal molecules to activate immune responses by interacting with metabotropic G-protein-coupled P2Y-receptors (P2Y_1-14_R) or the ionotropic P2X-receptors (P2X_1-7_R) in an autocrine or paracrine manner (11–13). P2Y1R is a Gα_q/11_ ADP receptor, which is abundant in nearly all human and rodent tissues (14). Besides its key role in the nervous system, P2Y1R also exerts potent immunity such as leading NLRP3 inflammasome priming and facilitating T cell activation in an ERK-dependent manner (13, 15). However, its function in regulation of Th17 cells is still unclear.

In this study, we found that the P2Y1R was upregulated in the peripheral lymphoid tissues of dextran sulfate sodium (DSS)-induced colitis mice and PMA and ionomycin-induced EL4 cells. Genetic deletion of P2Y1R attenuated the clinical symptoms of colitis, as well as suppressed Th17 differentiation *in vivo* and *in vitro*. Most importantly, we demonstrated that P2Y1R deficiency suppressing Th17 cell differentiation is dependent on AMPK activation.

## Materials and Methods

### Animals

Male C57BL/6 mice (Beijing Vital River Laboratory Animal Technology Co., Ltd., Beijing, China; certification: No.110322200101421145) at 6-8 weeks of age with weighing 18–22 g and P2Y1R^−/−^ C57BL/6 mice (Model animal research center of Nanjing University, Nanjing, China; certification: No.44007200071737) were used for the experiments (The genotyping was shown in **Supplementary Figure 1**). All the mice were kept in the environment without specific pathogen at the animal house of Shenzhen University and were fed with standard mouse feed and water *ad libitum*.

### Cell Culture and siRNA Transfection

The EL4 cells (Institute of Biochemistry and Cell Biology, Chinese Academy of Sciences, China) were cultured in Dulbecco’s modified eagle medium (DMEM; Invitrogen, Carlsbad, CA, USA) with 10% fetal bovine serum, 100 units/mL penicillin and 100 µg/mL streptomycin in a humidified atmosphere at 37°C with 5% CO_2_. All cells were passaged within 30 generations. Mouse AMPK targeted siRNA were obtained from Gene Pharma Company (Shanghai, China). The sequence of siRNA was listed as below: Sense 5′-GCCGACCCAAUGAUAUCAUTT-3′, antisense: 5′- AUGAUAUCAUUGGGUCGGCTT -3′. After EL4 cells were reached 60–70% confluence, AMPK targeted siRNA and control siRNA at 5 × 10^-8^ M were respectively transfected into EL4 cells using Lipofectamine 2000 reagent (Invitrogen, CA, USA) for 24 h, and then the cells were collected for analysis.

Primary naïve CD4^+^ T cells were magnetically isolated from mouse mesenteric lymph nodes using magnetic beads (Miltenyi Biotech, Cologne, Germany) binding to CD4^+^ T cells, and cultured in RPMI-1640 medium supplemented with 10% fetal bovine serum, 100 Units/mL penicillin and 100 µg/mL streptomycin.

### T Cell Activation

EL4 cells were seeded in a 96-well plate (1.5 × 10^4^ cells/well) overnight and then treated with 50 ng/mL phorbol myristate acetate (PMA; Sigma-Aldrich, St. Louis, USA) and 500 ng/mL ionomycin (Sigma-Aldrich, St. Louis, USA) for 6 h.

### Induction and Evaluation of Colitis

Mice were treated with 2% DSS (160110, molecular weight: 36,000–50,000 Daltons; MP Biologicals, LLC, France) drinking water for 5 days to induce colitis. Body weight and disease activity index (DAI) were measured and recorded every day. DAI was determined by percent of weight loss, degree of stool consistency and stool bleeding. At end of the experiment, the mice were sacrificed, and the length of the colon was determined. Subsequently, the colon sections were fixed in 4% paraformaldehyde for 24 h then embedded in paraffin, stained with hematoxylin-eosin (HE) (Sigma-Aldrich, St, Louis, MO, USA), and then evaluated and scored in a blind manner as our previously described (16, 17).

### Th17 Differentiation *In Vitro*

Primary CD4^+^ T cells were stimulated with an anti-CD3e (BD Pharmingen ™, USA) mAb (2 μg/mL) and anti-CD28 (BioLegend^®^, Germany) mAb (10 μg/mL) for 4 days under the following Th17 differentiation conditions: anti-IFN-γ (BioLegend^®^, Germany) (5 μg/mL), anti-IL-4 (BioLegend^®^, Germany) (5 μg/mL); anti-IL-23 (BioLegend^®^, Germany) (100 ng/mL), TGF-β (Miltenyi Biotech, Germany) (1 ng/mL), and IL-6 (BioLegend^®^, Germany) (40 ng/mL).

### Intracellular Staining

Polarized CD4^+^ T cells were primarily restimulated with PMA (50 ng/mL, Sigma-Aldrich, St. Louis, USA) and ionomycin (500 ng/mL, Sigma-Aldrich, St. Louis, USA) for 5h and then Brefeldin A (BioLegend^®^, Germany) was added 1h after PMA/Iono stimulation. Subsequently, cells were stained with anti-CD4 (Invitrogen, USA) and IL-17A (Invitrogen, USA) Ab for 45 min after blocking FcR, and then analyzed immediately by flow cytometry.

### RNA Extraction and Real-Time PCR

Total RNA was isolated and reversed using TRIzol^®^ (Thermo Fisher, USA) and 5X PrimeScript RT Master Mix (RR036A-1, TaKaRa; Shiga, Japan), respectively. Subsequently, the cDNA was analyzed by the ABI 7500 Real-Time PCR System with SYBR Green Master (Roche, Basel, Switzerland) for the expression of P2Y1R, IL-17A, RORγt, Foxp3, IFN-γ, GATA3, T-bet and IL-4 using the 2 ^−ΔΔ^CT method under the normalization of β-actin. Primer sequences (forward/reverse) of gene were listed in **Supplementary Table 1**.

### Western Blot Analysis

Western blot analysis were performed as our previously described (18). Used antibodies were listed as below: primary antibodies against p-AMPK (BS5003, Bioworld Technology, 1:1000), AMPK (BS1009, Bioworld Technology, 1:1000), RORγt (ab207082, abcam, 1:1000), GAPDH (2118S, Cell Signaling Technology, 1:1000) and anti-Rabbit IgG (7074S, Cell Signaling Technology, 1:2000). The fluorescent signals were visualized by ECL reagents and quantified using ImageJ software.

### ELISA Analysis and Myeloperoxidase (MPO) Determination

The extraction of the total proteins from colon samples was performed as described in our previous studies (16, 17). And then, the levels of the cytokines TNF-α, IL-6 and IL-17A in supernatants extracted from colon tissues and IL-6, IL-17A in the serum were determined according to manufacturer’s protocols (The ELISA kits of TNF-α, IL-6 and IL-17A used in the animal experiment of P2Y1R deficiency ameliorating murine colitis were obtained from JiangLai Biology, Shanghai, China, and ELISA kits of TNF-α, IL-6 and IL-17A used in the animal experiment of compound C intervention were obtained from Bioswamp, Wuhan, China). As well, the levels of MPO (JiangLai Biology, Shanghai, China) in supernatants extracted from colon tissues were also determined according to manufacturer’s protocols. The total protein in colon tissues sample was measured by the BCA protein assay kit (Solarbio, Beijing, China).

### Statistical Analysis

GraphPad Prism 5.0 software (GraphPad Software Inc., San Diego, CA, USA) was used to analyze the data. Results are expressed as mean ± standard error of the mean (SEM). Statistical significance between two groups was analyzed using the Student’s t-test. Differences among three or more groups were evaluated using one-way ANOVA, followed by Duncan’s multiple range tests. Probabilities of <0.05 were considered statistically significant.

## Results

### Increased P2Y1R Is Positively Associated With Th17 Response

**Figure 1:**
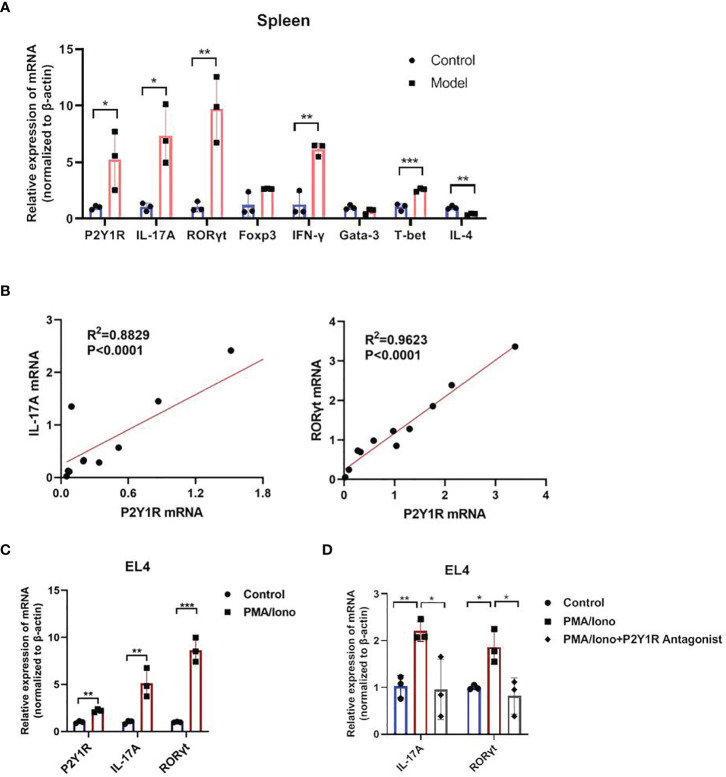
Increased P2Y1R is positively associated with Th17 response. **(A)** mRNA expression of P2Y1R, IL-17A, RORγt, Foxp3, T-bet, INF-γ, Gata-3 and IL-4 in the splenocytes of DSS-treated mice. Data are expressed as mean ± SEM (n=3). Compared to control mice, *p < 0.05, **p < 0.01 and ***p < 0.001. **(B)** Linear correlation analysis between transcripts of P2Y1R and IL-17A, RORγt in the splenocytes of DSS-treated mice (n=11). **(C)** mRNA expression of P2Y1R, IL-17A, RORγt in EL4 cells treated with PMA and ionomycin. **(D)** Antagonistic effect of P2Y1R on the gene expression of IL-17A and RORγt in EL4 cells treated with PMA and ionomycin. Data from EL4 cells are expressed as mean ± SEM of three independent experiments.

We firstly detected mRNA levels of P2Y1R and specific transcription factor and cytokines of Th1, Th2, Th17 and Treg in splenocytes of DSS-induced colitis in mice. The results showed that an increased P2Y1R expression in splenocytes of DSS-induced colitic mice compared to control littermates (**Figure 1A**). Interestingly, linear correlation analysis between transcripts of P2Y1R and specific transcription factor and cytokines of Th1 (T-bet and IFN-γ), Th2 (Gata-3 and IL-4), Th17 (RORγt and IL-17A) and Treg (Foxp3) showed that the expression of Th17 specific transcription factor RORγt and cytokine IL-17A correlated well with that of P2Y1R (**Figure 1B**), which were the best among the correlation of P2Y1R with other transcription factors and cytokines (**Supplementary Figure 2**). To explore the role of P2Y1R in regulation of Th17 cells, mRNA levels of P2Y1R was further detected in PMA and ionomycin-induced EL4 cells. As shown in **Figure 1C**, PMA and ionomycin challenge also increased the expression of P2Y1R as well as RORγt and IL-17A. Blockage of P2Y1R with MRS2179 (P2Y1R antagonist) significantly reversed PMA and ionomycin-induced the increase of RORγt and IL-17A expression (**Figures 1D**). These findings indicate that P2Y1R might be an important regulator of Th17 response in colonic inflammation.

### Deficiency of P2Y1R Ameliorates Murine Colitis and Its Th17 Responses

**Figure 2:**
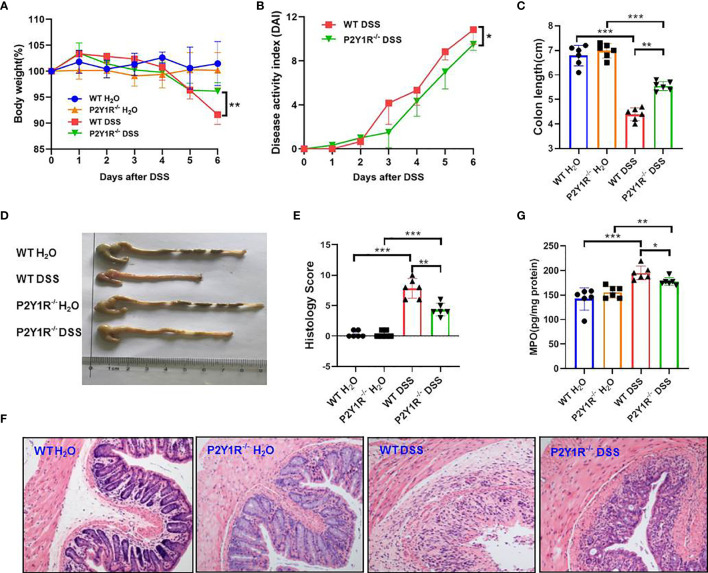
P2Y1R deficiency ameliorates DSS-induced colitis in mice. **(A)** Body weight change. **(B)** Disease activity index (DAI). **(C)** Colon length. **(D)** Representative photographs of colon. **(E)** Histopathological score of colon tissues. **(F)** Representative photographs of H&E staining (magnification × 200). **(G)** Myeloperoxidase (MPO) level. Data are expressed as mean ± SEM (n=6). *p < 0.05, **p < 0.01 and ***p < 0.001.

To verify the role of P2Y1R in regulation of Th17 cells in colonic inflammation, colitis of P2Y1R^-/-^ mice was induced by 2% DSS. As shown in **Figure 2**, compared to the DSS-treated WT controls, the body weight loss (at day 6), disease activity index (DAI), and colon length reduction were significantly alleviated in DSS-treated P2Y1R^-/-^ mice (**Figures 2A–D** and **Supplementary Figure 2**). Furthermore, histopathological analysis also showed that less inflammatory cell infiltration, crypt destruction and lesion formation, with lower scores of the histological changes were observed in DSS-treated P2Y1R^-/-^ mice (**Figures 2E, F** **)**. Consistent with the histological scores, colonic MPO level was also significantly decreased in DSS-treated P2Y1R^-/-^ mice (**Figure 2G**). These data indicate that P2Y1R deficiency significantly ameliorates DSS-induced murine colitis.

**Figure 3:**
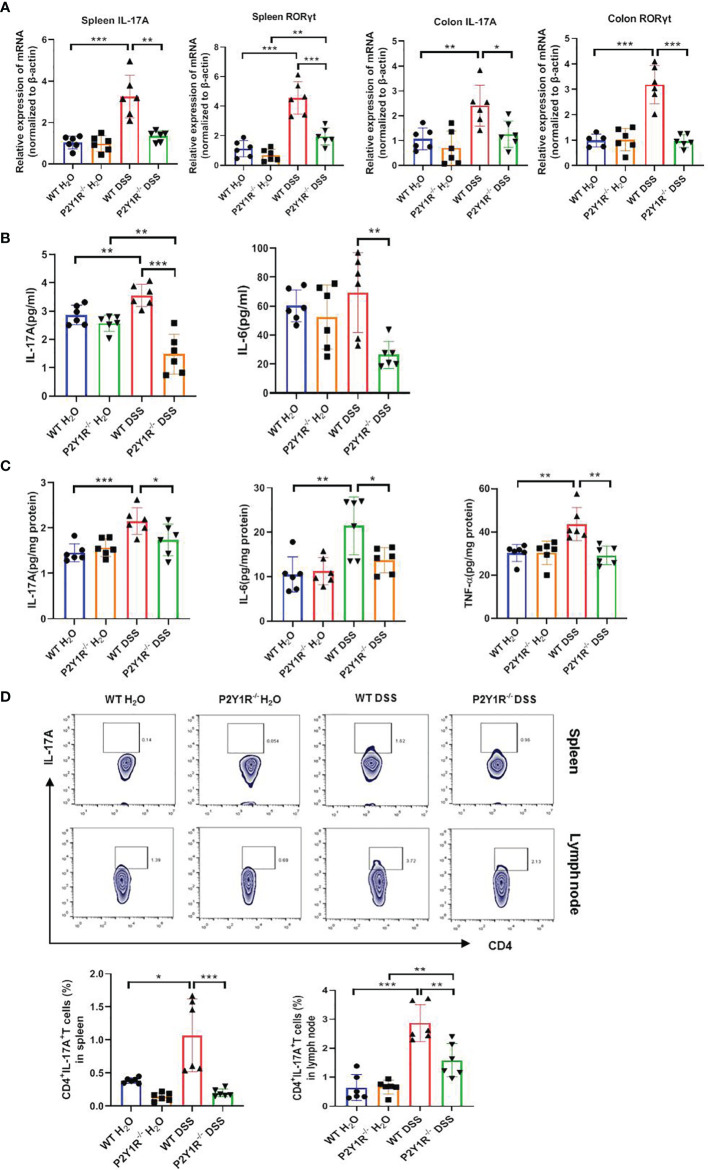
P2Y1R deficiency suppresses Th17 responses of DSS-treated mice. **(A)** mRNA expression of IL-17A and RORγt in the spleen and colon of DSS-treated P2Y1R^-/-^ and WT mice. **(B)** Levels of proinflammatory cytokines IL-6 and IL-17A in the serum of DSS-treated P2Y1R^-/-^ and WT mice. **(C)** Levels of proinflammatory cytokines IL-6, IL-17A and TNF-α in the colon tissues of DSS-treated P2Y1R^-/-^ and WT mice. **(D)** The frequency of Th17 cells in the spleen and mesenteric lymph nodes (MLN) of both DSS-treated P2Y1R^-/-^ and WT mice. Data are expressed as mean ± SEM (n=5-6). *p < 0.05, **p < 0.01 and ***p < 0.001.

Subsequently, Th17-specific transcription factor and cytokines were assessed in both DSS-treated P2Y1R^-/-^ and WT mice. As showed in **Figure 3A**, relative mRNA levels of Th17-specific transcription factor RORγt and proinflammatory cytokine IL-17A were downregulated significantly in the spleen and colon tissues of DSS-treated P2Y1R^-/-^ mice versus WT controls. In parallel, compared to the DSS-treated WT controls, decreased protein levels of IL-6, IL-17A in the serum as well as decreased protein levels of IL-6, IL-17A and TNF-α in the colon were also observed in DSS-treated P2Y1R^-/-^ mice (**Figures 3B, C** **)**. Moreover, we analyzed the proportion of Th17 cells in the spleen and mesenteric lymphoid nodes (MLN) of both DSS-treated P2Y1R^-/-^ and WT mice. As expected, the population of Th17 cells in DSS-treated P2Y1R^-/-^ mice was significantly decreased in comparison of their WT controls (**Figure 3D**). These data indicate that P2Y1R deficiency significantly suppresses Th17 responses in mice with DSS-induced colitis.

### Deficiency of P2Y1R Impairs Th17 Cell Differentiation *via* AMPK Activation

**Figure 4:**
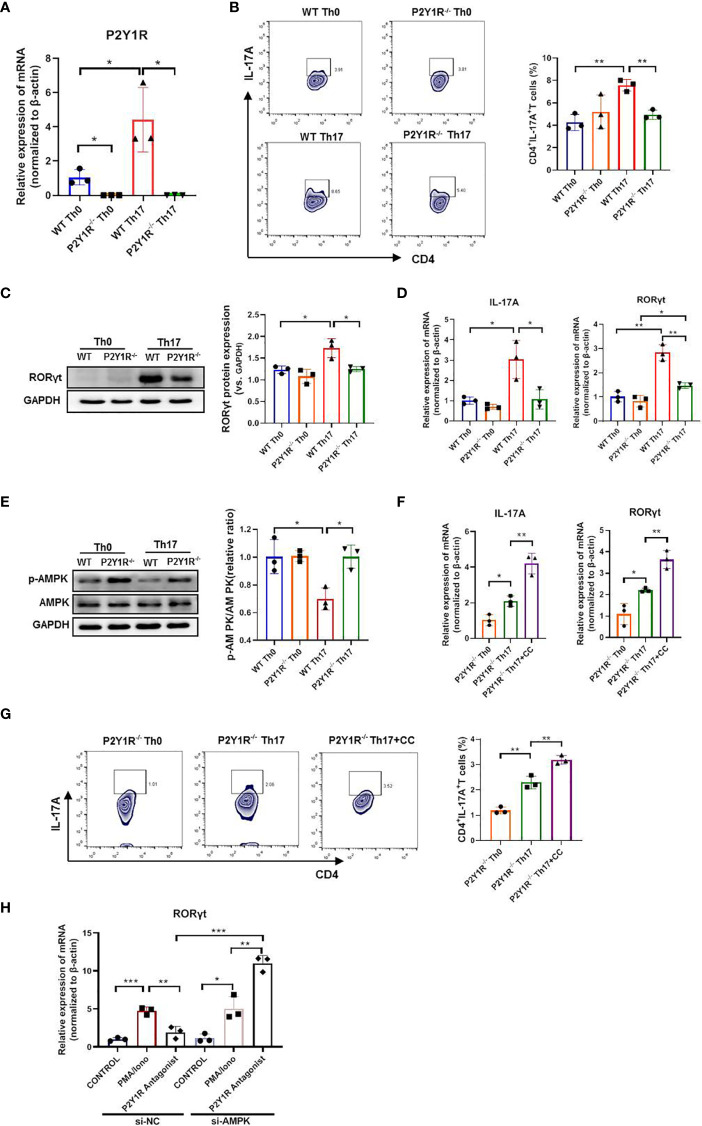
Deficiency of P2Y1R impairs Th17 cell differentiation *via* AMPK activation *in vitro*. **(A)** mRNA expression of P2Y1R on Th0 and Th17 polarized CD4^+^ T cells derived from P2Y1R^-/-^ mice and WT mice. **(B)** P2Y1R deficiency suppresses Th17 differentiation *in vitro*. **(C)** P2Y1R deficiency decreases protein expression of RORγt in Th17 polarized CD4^+^ T cells. **(D)** P2Y1R deficiency decreases the mRNA expression of RORγt and IL-17A in Th17 polarized CD4^+^ T cells. **(E)** P2Y1R deficiency induces AMPK phosphorylation of Th17 polarized CD4^+^ T cells. **(F)** Blockage of AMPK increases the mRNA expression of RORγt and IL-17A in Th17 polarized CD4^+^ T cells derived from P2Y1R^-/-^ mice. **(G)** Blockage of AMPK increases Th17 differentiation of naïve CD4^+^ T cells derived from P2Y1R^-/-^ mice *in vitro*. **(H)** Silence of AMPK in PMA and ionomycin-induced EL4 cells reversed the inhibitory effect of P2Y1R antagonist MRS2179 on RORγt mRNA expression. Data from are expressed as mean ± SEM of three independent experiments. *p < 0.05, **p < 0.01 and ***p < 0.001.

To determine whether P2Y1R directly regulated Th17 cell generation, we performed an *in vitro* T-cell differentiation assay. We first examined the P2Y1R expression on naïve CD4^+^ T cells under Th17-skewing conditions or not, as shown in **Figure 4A**, the mRNA expressions of P2Y1R in P2Y1R^-/-^ Th0 and Th17 cells were absent, and mRNA expressions of P2Y1R in WT-Th17 cells was greatly increased. Subsequently, we tested the effect of P2Y1R deficiency on Th17 cell differentiation. As shown in **Figures 4B–D**, under Th17-skewing conditions, P2Y1R deficiency decreased the proportion of Th17 cells, as well as the gene expressions of RORγt and IL-17A were also greatly decreased. Concurrently, the protein expression of RORγt in naïve CD4^+^ T cells under Th17-skewing conditions was also decreased after P2Y1R deficiency. We next tried to find out the molecular mechanisms by which P2Y1R deficiency regulates the differentiation of Th17 cells. Based on previous findings that P2Y1R might mediate inhibition of AMPK (19), which in turn could regulate the differentiation of Th17 cells (20). We examined the phosphorylation of AMPK in naïve CD4^+^ T cells under Th17-polarizing conditions. Interestingly, AMPK phosphorylation in polarized CD4^+^ T cells derived from P2Y1R^-/-^ mice was significantly increased (**Figure 4E**). Addition of AMPK inhibitor (Compound C, CC, 50 μM; Sigma-Aldrich, St. Louis, USA) significantly reversed the inhibitory effect of P2Y1R deficiency on Th17 cell differentiation (**Figures 4F, G** **)**. As well, silence of AMPK in PMA and ionomycin-induced EL4 cells also notably reversed the inhibitory effect of P2Y1R antagonist MRS2179 on RORγt mRNA expression (**Figures 4H**). These findings indicate that P2Y1R deficiency impairs Th17 cell differentiation through AMPK activation.

### Blocking the AMPK Pathway Attenuates the Anti-Colitis Effect Conferred by P2Y1R Deficiency in Interfering Th17 Responses

**Figure 5:**
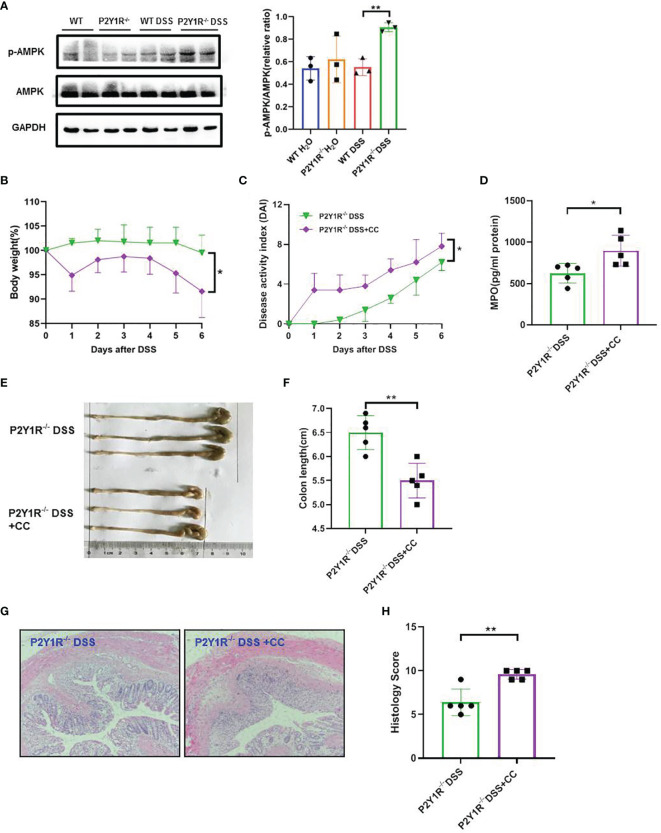
Blockage of AMPK worsens the severity of colitis in DSS-treated P2Y1R^-/-^ mice. **(A)** P2Y1R deficiency induces AMPK phosphorylation in the colon of DSS-treated mice (n=3). **(B)** Body weight change. **(C)** Disease activity index (DAI). **(D)** Myeloperoxidase (MPO) level. **(E)** Representative photographs of colon. **(F)** Colon length. **(G)** Representative photographs of H&E staining (magnification × 200). **(H)** Histopathological score of colon tissues. Data are expressed as mean ± SEM (n=5). *p < 0.05 and **p < 0.01.

**Figure 6:**
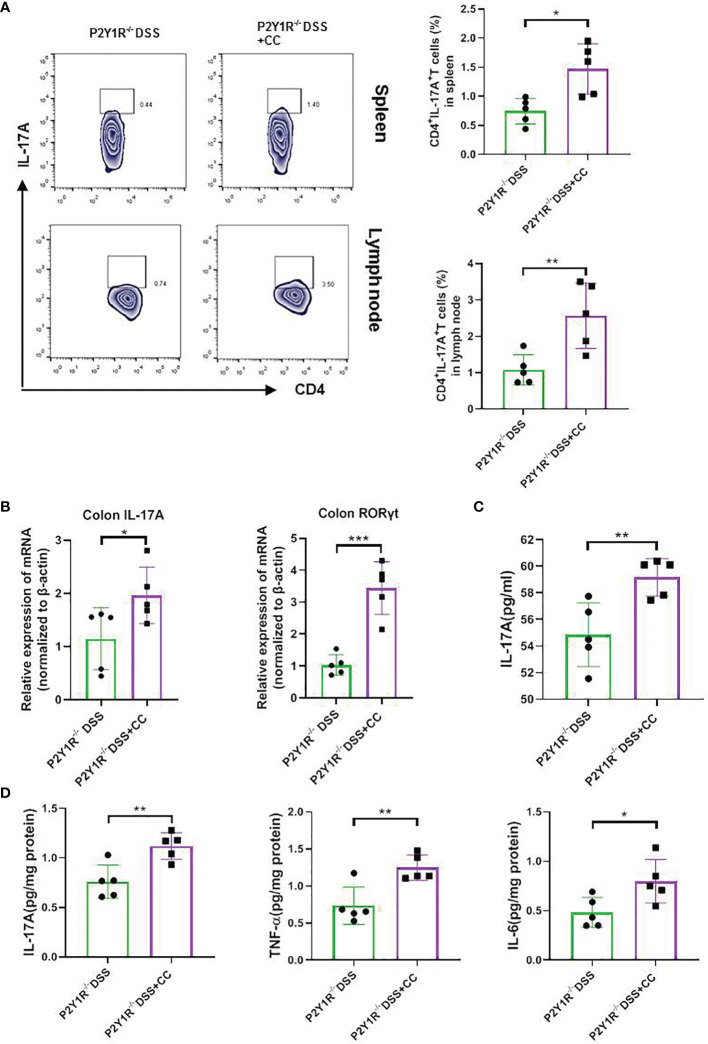
Blockage of AMPK increased the Th17 responses of DSS-treated P2Y1R^-/-^ mice. **(A)** The frequency of Th17 cells in the spleen and mesenteric lymph nodes (MLN) of DSS-treated P2Y1R^-/-^ mice. **(B)** mRNA expression of IL-17A and RORγt in the colon of DSS-treated P2Y1R^-/-^ mice. **(C)** Levels of proinflammatory cytokines IL-17A in the serum of DSS-treated P2Y1R^-/-^ mice. **(D)** Levels of proinflammatory cytokines IL-6, IL-17A and TNF-α in the colon tissues of DSS-treated P2Y1R^-/-^ mice. Data are expressed as mean ± SEM (n=5). *p < 0.05, **p < 0.01 and ***p < 0.001.

To further investigate whether the effects of P2Y1R deficiency take by AMPK activation *in vivo*, we first examined the expression of AMPK phosphorylation in the colon tissues of colitis mice and found that p-AMPK was higher expressed in the colon tissues of colitic P2Y1R^-/-^ mice in comparison of colitic WT mice (**Figure 5A**). As well, we also evaluated the efficacy of the AMPK antagonist Compound C (CC, 10 mg/Kg, intraperitoneal injection; 171261, Sigma-Aldrich, St. Louis, USA) in P2Y1R^-/-^ mice with colitis. As shown in **Figures 5B–H**, treatment of recipients with Compound C resulted in increased body weight lost, DAI score, colon shorten, colonic MPO level and colonic injury. As P2Y1R deficiency-mediated altered Th17 cell differentiation occurred *via* AMPK activation *in vitro*, we further assessed whether the Th17 responses in P2Y1R^-/-^ mice with colitis was influenced by the AMPK antagonist Compound C. Compared to DSS-treated P2Y1R^-/-^ control mice, the proportion of Th17 in the spleen was significantly increased after Compound C treatment (**Figure 6A**). Consistent with this result, Compound C increased gene expression of IL-17A and RORγt in the colon tissues, and also increased protein level of IL-17A levels in the serum and protein levels of IL-6, IL-17A and TNF-α in the colon tissues compared to those in DSS-treated P2Y1R^-/-^ control mice (**Figures 6B–D**). Collectively, these findings indicated that blocking the AMPK pathway impaired the protective effect conferred by P2Y1R deficiency *via* interfering with Th17 response in experimental colitis.

## Discussion

Th17 cells have been involved in the pathogenesis of IBD. To find effective ways to selectively inhibit the development and function of Th17 cells means a reasonable strategy to cure this disease (21, 22). In this study, we clearly indicated that the P2Y1R is an important regulator to control Th17 cell generation, and targeting P2Y1R is a potential therapeutic strategy for the treatment of IBD, as evidenced by deleting of P2Y1R could impair Th17 cell differentiation *in vitro* and ameliorate mouse colitis and its Th17 responses *in vivo*. Moreover, we demonstrated that the inhibitory effect of P2Y1R deficiency on Th17 cell differentiation is dependent on AMPK activation.

P2Y1R, as one of G protein-coupled receptors, is expressed in lymphoid tissues such as thymus, spleen and lymph nodes where they are expressed on macrophages, eosinophils, neutrophils, monocyte-derived DCs, B and T cells (23). Previous studies have indicated that P2Y1R is a key player in the innate immunity, which could regulate the chemotaxis and reactive oxygen metabolites production of eosinophils (23), the capacity of monocyte-derived DCs to attract monocytes (24), the phagocytic ability of macrophages (25), histamine release from mast cells (26). Recently, Woehrle T et al. found that P2Y1R could regulate T cell function. It found that exogenous ADP or overexpression of P2Y1R could dramatically increase mRNA transcription of IL-2 in response to TCR/CD28 stimulation, and antagonists or silencing of P2Y1R could reduce mRNA transcription of IL-2 and mitochondrial Ca^2+^ uptake in stimulated CD4^+^ T cells (15). However, the role of P2Y1R in regulation of adaptive immunity still remains largely unexplored. In the present study, we detected that increased P2Y1R was expressed in splenocytes of DSS-treated mice and PMA and ionomycin-induced EL4 cells, and firstly found that increased P2Y1R was positively associated with the mRNA expression of RORγt and IL-17A in DSS-treated mice. Antagonist of P2Y1R could reduce RORγt and IL-17A expression in PMA and ionomycin-induced EL4 cells. These findings indicated that P2Y1R could directly regulate Th17 responses. Subsequently, we used P2Y1R knockout mice to verify that P2Y1R deficiency significantly ameliorated colitis and its Th17 responses, and P2Y1R deficiency could also greatly impaired Th17 cell differentiation *in vitro*. Collectively, these data clearly demonstrated that inhibition of P2Y1R could directly suppress Th17 cell differentiation to ameliorate experimental colitis and suggested that P2Y1R is a potential target to control Th17 cell generation.

How P2Y1R modulate Th17-cell differentiation? This remains to be explored. The AMP-activated protein kinase (AMPK) is a key regulator of metabolism existed in virtually all eukaryotic cells (27). Cui J et al. reported that P2Y1R is essential to astrocyte activation in spinal cord, which could mediate inhibition of AMPK to suppress over activation of the astrocytes, which induced by extracellular ADP (19). Notably, several lines of evidence have documented that AMPK plays a decisive role in regulation of Th17 cell generation. It has been reported that AMPK activator AICAR (5-aminoimidazole-4-carboxamide ribonucleotide) could effectively inhibit Th17 differentiation (28), and AMPK inhibitor Compound C could promote Th17 differentiation (29, 30). As well, Kang KY et al. also reported that AMPK activator metformin significantly inhibit Th17 cell differentiation to attenuate murine autoimmune arthritis (31). We therefore determinated the AMPK phosphorylation in polarized CD4^+^ T cells under Th17-skewing conditions, and found that AMPK phosphorylation in polarized CD4^+^ T cells derived from P2Y1R^-/-^ mice was significantly increased. As well, the same increased AMPK phosphorylation was also observed in the colon of DSS-treated P2Y1R^-/-^ mice in comparison with their WT controls. Antagonist of AMPK significantly reversed the inhibitory effect of P2Y1R deficiency on Th17 cell differentiation *in vivo* and *in vitro*. These findings clearly demonstrated that P2Y1R modulates the differentiation of Th17 cells in an AMPK -dependent manner.

## Conclusions

In summary, the current study demonstrated that P2Y1R deficiency could suppress Th17 cell differentiation to ameliorate IBD in an AMPK -dependent manner, and proved that targeting to P2Y1R had potential application value in the treatment of IBD.

## Data Availability Statement

Raw data of all figures (including original files of flow cytometry and images of HE staining, and full length, uncropped western blot images) were uploaded in Jianguoyun, please review them from the link: https://www.jianguoyun.com/p/DYaQD9MQ2byBChjXrp0E (Access password : nmkmxm).

## Ethics Statement

The animal study was reviewed and approved by Animal Ethics Committees of Shenzhen University, China.

## Author Contributions

H-TX: Conceptualization, investigation, writing-original draft and funding acquisition. Y-YC and Q-CH: Investigation. JP: Investigation and funding acquisition. W-CB and L-XZ: Data analysis, methodology and resources. YC, JL, and Z-XB: Conceptualization and edited the manuscript. H-qW, Y-XC, and X-CS: Supervision, methodology and resources. All authors contributed to the article and approved the submitted version.

## Funding

This work was kindly funded by the National Natural Science Foundation of China (81560676 and 81660479), SZU Top Ranking Project (86000000210) and Foundations of Shenzhen Science and Technology Innovation Committee (JCYJ20190808164201654 and JCYJ20210324093810026), Medical Science and Technology Research Foundation of Guangdong province, China (A2020157 and A2020272).

## Conflict of Interest

The authors declare that the research was conducted in the absence of any commercial or financial relationships that could be construed as a potential conflict of interest.

## Publisher’s Note

All claims expressed in this article are solely those of the authors and do not necessarily represent those of their affiliated organizations, or those of the publisher, the editors and the reviewers. Any product that may be evaluated in this article, or claim that may be made by its manufacturer, is not guaranteed or endorsed by the publisher.

## Abbreviations

ADP, Adenosine diphosphate; AICAR, 5-aminoimidazole-4-carboxamide ribonucleotide; AMPK, AMP-activated protein kinase; ATP, Adenosine triphosphate; CD, Crohn’s disease; CC, Dorsomorphin (Compound C); DAI, Disease activity index; DSS, Dextran sulfate sodium; H&E, Hematoxylin and eosin; IBD, Inflammatory bowel disease; Iono, Ionomycin; MPO, Myeloperoxidase; PMA, Phorbol myristate acetate; P2Y1R, P2Y1 receptor; RORγt, Retineic-acid-receptor-related orphan nuclear receptor gamma; UC, Ulcerative colitis; WT, Wildtype.
