# Supplementary material for: P2Y1R Ligation Suppresses Th17 Cell Differentiation and Alleviates Colonic Inflammation in an AMPK-Dependent Manner
Source: Front Immunol. 2022 Feb 10;13:820524. doi: 10.3389/fimmu.2022.820524 (PMC8866175; doi:10.3389/fimmu.2022.820524)
Supplement: Supplementary file 1 [file DataSheet_1.docx]

**P2Y1R ligation suppresses Th17 cell differentiation and alleviates colonic inflammation in an AMPK-dependent manner**

Yao-Yao Chang^1*^, Qiu-Chan Huan^2,3*^, Jiao Peng^3*^, Wen-Chun Bi^1^, Li-Xiang Zhai^4^, Yan Chen^5^, Jonathan R Lamb^6^, Xiang-Chun Shen^2^, Zhao-Xiang Bian^4^, Yong-Xian Cheng^1^, Hai-Tao Xiao^1*^

^1^School of Pharmaceutical Sciences, Health Science Center, Shenzhen University, Shenzhen, China.

^2^The State Key Laboratory of Functions and Applications of Medicinal Plants and The High Efficacy Application of Natural Medicinal Resources Engineering Center of Guizhou Province, School of Pharmaceutical Sciences, Guizhou Medical University, Guizhou, China. ^3^Department of Pharmacy, Peking University Shenzhen Hospital, Shenzhen, China.

^4^School of Chinese Medicine, Hong Kong Baptist University, Kowloon, Hong Kong

^5^Department of Surgery, the University of Hong Kong, Hong Kong.

^6^Department of Life Sciences, Faculty of Natural Sciences, Imperial College London, London, United Kingdom.

*These authors have contributed equally to this work.

**Correspondence:**

Hai-Tao Xiao, Ph.D. School of Pharmaceutical Sciences, Health Science Center, Shenzhen University, 518060, Shenzhen, China. Email: [xhaitao@szu.edu.cn](mailto:xhaitao@szu.edu.cn)

1. **Genotyping of P2Y1R^-/-^ mice**

**1.1 Genomic DNA extraction:** Each 0.5 cm mouse tail was cut into a 2 mL EP tube respectively, and then 475 μL lysate (c500028-0010, BBI, Shanghai, China) and 2.5 μL proteinase K (ST535, Beyotime, Shanghai, China) (20 μg/μL) were added to each tube and mixed well. Subsequently, the tubes were put on a constant temperature electric heating shaking box to shock overnight at 55°C. And then, the tubes were centrifuged at 12,000 rpm at 4℃ for 10 min, the upper layer of each tube was transferred to another new EP tube, respectively. Next, an equal volume of isopropanol (B2101071, Xilong Science, China) was added to each tube and mixed well, and then the mixtures were centrifuged at 1200 rpm at 4℃ for 10 min. The upper layer of each tube was discarded and 1 mL 75% ethanol was added respectively and mixed well, and then the mixture were centrifuged at 8000 rpm at 4℃ for 10 min. After discarding the ethanol, the DNA was dried in a sterile condition, and then 200 μL ddH2O was added to measure the concentration immediately.

**1.2 PCR reaction:** Primer sequences: P2Y1R-KO, forward 5′- CAG AAT GTG GCC GGA AGA AGA G- 3′ and reverse 5′- GCG ACC ATG TAT GCT TCT GAG G- 3′, the length of the target gene fragment is 675bp; WT, forward 5′- TTC CAC ATG AAG CCT TGG AGC G - 3′ and reverse 5′- CAC CAA AGG GAT GCA GAA CAT GG - 3′, the length of the target gene fragment is 462bp.

**1.3 Reaction system:** 9.5 μL ddH_2_O, 12.5μL 2×Taq Master Mix (71572D1, Vazyme, Nanjing, China), 0.5μL WT-F, 0.5μL WT-R, 0.5μL P2Y1R-KO-F, 0.5 μL P2Y1R-KO-R, 1μL DNA template, 20μL in total.

**1.4 Reaction conditions:** pre-denaturation: 94 ℃, 5min; denaturation: 94 ℃, 20s; annealing: 60 ℃, 30s; extension: 72 ℃, 30s, 35 cycles in total; final extension: 72 ℃, 5 min, storage at 4 ℃.

**1.5 2% agarose gel electrophoresis:** 0.75g Agarose B, Low EEO (A600014-0250, BBI, Shanghai, China), 50 mL 1× TAE (ST716, Beyotime, Shanghai, China), 5μL 4S Green Plus Nucleic Acid Stain (A616696-0100, BBI, Shanghai, China); loading: 10μL reaction system. Voltage: 110 V; time: 30 min.

**1.6 The results of genotyping by PCR**

**
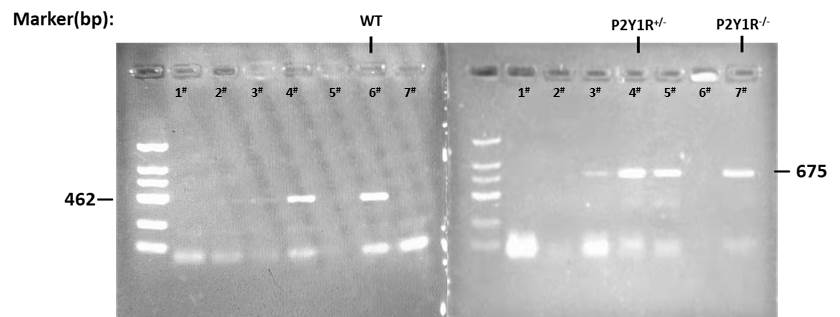
**

**Figure S1** Genotyping of P2Y1R^-/-^ mice

1. **Linear correlation of P2Y1R and specific transcription factor and cytokines of Th1 (T-bet and IFN-γ), Th2 (Gata-3 and IL-4) and Treg (Foxp3)**


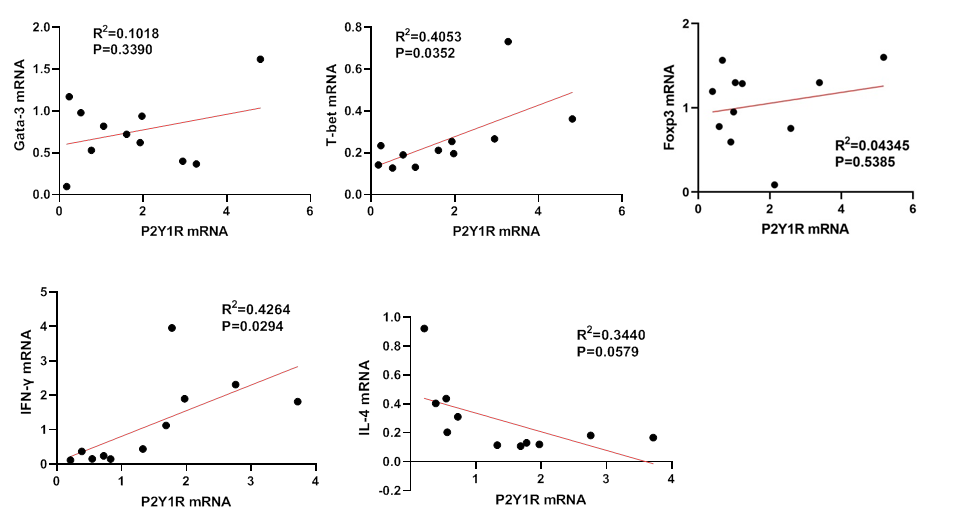


**Figure S2** Linear correlation analysis between transcripts of P2Y1R and specific transcription factor and cytokines of Th1 (T-bet and IFN-γ), Th2 (Gata-3 and IL-4) and Treg (Foxp3) in splenocytes of DSS-induced colitis in mice (n=11).

**Table S1 Primer sequences of genes**

| Name | Sequence |
| --- | --- |
| Mouse IL-17A | Forward 5′-TTTAACTCCCTTGGCGCAAAA-3′ |
|  | Reverse 5′- CTTTCCCTCCGCATTGACAC- 3′ |
| Mouse RORγt | Forward 5′-GACCCACACCTCACAAATTGA-3′ |
|  | Reverse 5′- AGTAGGCCACATTACACTGCT-3′ |
| Mouse Foxp3 | Reverse 5′- CCCATCCCCAGGAGTCTTG-3′ |
|  | Reverse 5′- ACCATGACTAGGGGCACTGTA-3′ |
| Mouse IFN-γ | Reverse 5′-ATGAACGCTACACACTGCATC-3′ |
|  | Reverse 5′-CCATCCTTTTGCCAGTTCCTC-3′ |
| Mouse T-bet | Reverse 5′-GATCACTCAGCTGAAAATCGAC-3′ |
|  | Reverse 5′-AGGCTGTGAGATCATATCCTTG-3′ |
| Mouse Gata-3 | Reverse 5′-ATTACCACCTATCCGCCCTAT-3′ |
|  | Reverse 5′-CGGTTCTGCCCATTCATTTTAT-3′ |
| Mouse IL-4 | Reverse 5′-GGTCTCAACCCCCAGCTAGT-3′ |
|  | Reverse 5′-GCCGATGATCTCTCTCAAGTGAT-3′ |
| Mouse P2Y1R | Reverse 5′-GGGCTCGCTTTGGGGAAACAG-3′ |
|  | Reverse 5′-CGGTCTTGGTCAGGGCACATTG-3′ |
| Mouse β-actin | Reverse 5′-GGCTGTATTCCCCTCCATCG-3′ |
|  | Reverse 5′-CCAGTTGGTAACAATGCCATGT-3′ |
