# Supplementary figures and images for: P2Y1R Ligation Suppresses Th17 Cell Differentiation and Alleviates Colonic Inflammation in an AMPK-Dependent Manner
Source: Front Immunol. 2022 Feb 10;13:820524. doi: 10.3389/fimmu.2022.820524 (PMC8866175; doi:10.3389/fimmu.2022.820524)

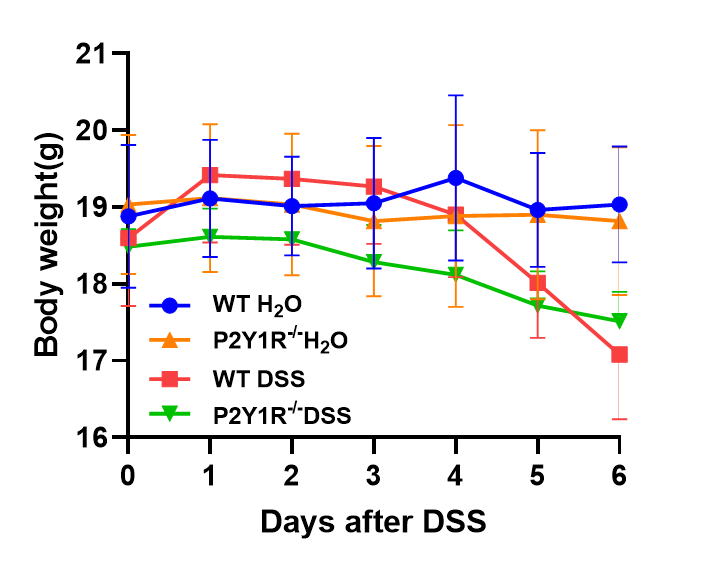

Supplement: Supplementary file 2 [file Image_1.png]
